# Adolescents living with HIV, complex needs and resilience in Blantyre, Malawi

**DOI:** 10.1186/s12981-020-00292-1

**Published:** 2020-06-22

**Authors:** Blessings N. Kaunda-Khangamwa, Prosperina Kapwata, Kennedy Malisita, Alister Munthali, Effie Chipeta, Sam Phiri, Lenore Manderson

**Affiliations:** 1 School of Public Health, The University of the Witwatersrand, Wits Education Campus. 27 St. Andrews Road, Parktown, Johannesburg, 2193 South Africa; 2 The School of Public Health and Family Medicine, University of Malawi, College of Medicine, Blantyre, Malawi; 3 The Malaria Alert Centre, University of Malawi, College of Medicine, Blantyre, Malawi; 4 Umodzi Family Centre, Lighthouse Trust, Blantyre, Malawi; 5 Centre for Social Research, University of Malawi, Chancellor College, Zomba, Malawi; 6 Centre for Reproductive Health, University of Malawi, College of Medicine, Blantyre, Malawi; 7 Lighthouse Trust, Lilongwe, Malawi; 8 School of Social Sciences, Monash University, Melbourne, Australia; 9 Institute at Brown for Environment and Society, Brown University, Providence, RI USA

**Keywords:** Adolescents living with HIV, Complex needs, Resilience, Differentiated care, HIV, Malawi, Services, Teen clubs

## Abstract

**Background:**

Adolescents living with HIV (ALHIV) in Malawi experience multiple challenges associated with their illness and various social, environmental, economic and cultural factors. In exploring their various medical concerns and social vulnerabilities, we consider the role of multiple services in creating a pathway for resilience.

**Methods:**

Multiple methods and case studies allowed for triangulation of evidence and provided a holistic understanding of resilience among adolescents with complex needs. The research methods included: (1) a survey to identify examples of young people with complex needs, (2) qualitative interviews and field notes to further explore these needs, (3) patient files and health passports to identify clinical challenges, and (4) ecomapping exercises to personalize cases and identify resilience-enabling resources and supports. We present four case studies to highlight the complex experiences and access to services of ALHIV, and to illustrate their growing power and decision-making capacity over time.

**Results:**

Adversity experienced by ALHIV varied by gender, family situation, years of schooling, and use of teen-clubs for support. The two female adolescents emphasised their need to be accepted and how this impacted sexuality and reproduction. The two males illustrated how ideas of masculinity influenced their sexual practice and involvement with health services and the correctional justice system. Multiple risks (alcohol use, sexual activities) and complex needs (belonging, having a purpose in life/productive activities, autonomy, desire for offspring) influence pathways to resilience. ALHIV were able to strengthen their own wellbeing by resisting negative behaviours and peer pressure and caregiver interactions through ‘strategic silence’.

**Conclusion:**

ALHIV experienced self-transformation as a result of taking ART, with fewer severe episodes of illness and distressing skin conditions. Continuous engagement at the teen-club clinic transformed both productive activities and social relationships among ALHIV as they set life goals, gained a sense of empowerment, requested SRH services, and formed intimate relationships. These transformative opportunities allowed them to learn ways of minimizing risk of reinfection and violence, and of navigating health worker–caregiver–adolescent interactions.

## Introduction

Many adolescents, globally, experience poverty, abuse, learning difficulties, family breakdown, stigma and discrimination [1–3]. Infection with HIV compounds these disadvantages. Compared with peers of the same age without a chronic illness, ALHIV have difficulties economically, socially and psychologically. This leads to low aspirations, poor goals and a sense of failure [4–6]. A culture of silence around HIV and sexual reproductive health (SRH) issues is still dominant, and is at times strategic to avoid exclusion [6–9]. In this article, we used a case study approach to identify the complex characteristics of ALHIV in Malawi to explain how young people manage their lives in the face of adversity.

Poor access to contraception exposes adolescents to unintended pregnancies and sexually transmitted infections (STIs) including HIV. Fifty percent of births in sub-Saharan Africa are among women aged 15–19 years, and early pregnancies and births reflect early sexual debut and low contraception use [10–12]. Unintended early pregnancy affects the physical maturation of young women, and can result in forced marriage, disruptions to education, and mother-to-child HIV transmission [12, 13]. Continuing social stigmatisation may influence young women to seek an unsafe abortion and risk death to avoid community ostracism [14, 15], while stigma also impacts directly on adolescents—male and female—with HIV [13]. HIV/AIDS is the second leading cause of death among adolescents aged 10–19 globally and in sub-Saharan Africa [13, 16]. However, early death from HIV is no longer inevitable.

To understand how adolescents’ navigate and negotiate the ‘complex mix’ of social, biological, physical and cultural experiences for their wellbeing, we adopt a complex needs perspective. Most studies on complex needs focus on people with mental illness, who have experienced substance or sexual abuse, learning difficulties, physical challenges, poverty or social exclusion [17–19], and so tie ‘complex needs’ both to the disadvantaged circumstances of clients and the difficulties service providers face in working with them [20]. These issues are significant for ALHIV as they negotiate the complexities of their personal and social lives, as Rankin and Regan [17] suggest:

Understanding complex needs is important in several senses. It allows practitioners to explore the breadth and depth of people’s needs and the interaction between different needs. It also highlights a severely excluded group: people who require a more carefully targeted approach from universal services [17].

Ungar and colleagues [21] use the terms ‘complex needs’ and ‘multiple service-using youths’ interchangeably to capture the challenges of adolescents in accessing education, and interacting with child welfare, correctional and mental health services. They argue that because the provision of psychosocial services is delivered in departmental silos, there is a lack of information flow, poor intrapersonal relationships, and structural boundaries. This results in poor services and care for young people [16, 21], and has led to the increasing trend for ‘bundled’ health care programmes, e.g., ‘family support, psychosocial support and SRH services’ [3], or ‘minimum health package for STIs, HIV and AIDS, family planning information and services, nutrition, sexual abuse, adolescent pregnancy, and psychosocial support’ [20, 22]. A combination of acceptable and clinically scalable health-care packages that address social and behavioural factors, complemented with structural support, are considered to have a maximum impact [3, 22, 23]. This is true in low and middle-income countries as well as high-income settings.

In Malawi, young people use medical and social services with mixed outcomes [18, 24–28]. Medical, biological and social factors influence the complexity of diseases and wellbeing over time, with interactions across these factors influencing health-seeking and outcomes [29–31]. The complex mix of social and biological factors is conceptualised as a syndemic, in which there is often stress both as a cause and as the result of physical health problems [31, 32]. Structural disadvantage and vulnerability, family fragmentation, extreme poverty, food insecurity, lack of shelter and interpersonal violence further shape risk and outcome. Our interest in this article is in how to intervene. We turn to a teen-club clinic model in Malawi [1, 33]. Drawing on case studies, we consider how disease (including TB, STIs, HIV), structural factors (gender, class, poverty, education) and social forces (child-headed homes or correctional/juvenile centres, unintended pregnancy) complicate the wellbeing of ALHIV [13, 23, 25, 34].

### Resilience, socio-ecological framework and ALHIV

Resilience is defined as the capacity of individuals to navigate their way to the psychological, social, cultural, and physical resources that sustain their well-being, and their capacity individually and collectively to negotiate for these resources to be provided in culturally meaningful ways [35]. The concept of resilience highlights the interactions between protective and constraining factors and processes that need to be considered for adolescents to do well despite adversity [13, 36]. Ungar’s definition is grounded in a socio-ecological understanding of resilience. Both resilience and ecological approaches center on the complexity of processes and, interactions, with a shift of focus from individual traits, relational, community to institutional support [35, 37]. We use resilience as a strength-based perspective to enable insights into positive outcomes among ALHIV and to counter dominant negative narratives of ALHIV. In describing this, we illustrate how attention to resilience establishes space to identify unique perspectives and to give voice to ALHIV in Malawi [38].

## Methods

### Study design

In this study, we used a multiple methods approach with case studies to examine the experiences of ALHIV as influenced by age, gender and the utilisation (or not) of health services [39, 40]. We created narratives of these adolescents accessing services to gain an understanding of their lives. Data collection included the administration of the Child Youth Resilience Measure-28 (CYRM-28) to identify adolescents’ complex needs and service usage, recorded qualitative in-depth interviews, and field notes. These data were supplemented by a review of patient files and health passports to situate clinical challenges, and an ecomapping exercise to personalise experiences and to strengthen diagrammatic representations of social and health services utilisation over time [41, 42].

### Setting

The study from which the cases were drawn was conducted at an ART and teen-club clinic in Blantyre [43]. This city has a population of about 800,000 people and is described as a hotspot for HIV, with a prevalence of 18% among persons aged 15–49 [43, 44]. The teen club was set up in 2010 and is currently run by Lighthouse Trust at the Umodzi Family Care (UFC) clinic at the Queen Elizabeth Central Hospital (QECH) in Blantyre. The teen club provides adolescent-centred psychosocial support to ALHIV, with its program adapted from the Baylor College of Medicine (USA) International Paediatric AIDS Initiative Centre of Excellence Curriculum [1]. The club had a membership of 3500 young people aged 10–24 as of June 2019, and serves as a one-stop-shop offering comprehensive HIV care and supports including SRH, nutrition, education and entrepreneurship services for ALHIV. Other services are prevention, care and treatment for other STIs (such as gonorrhoea, syphilis and genital warts), targeted family planning, teachings on sexuality, and basic computer skills. The 10–19 year olds meet monthly, while the transitioned teens (usually 20 years and above) meet once every quarter of a year.

### Sampling and participation

Purposive sampling with maximum variation was used to select adolescents who self-identified as having experienced multiple or complex health and social problems. We explained to them that we wished to learn about their lives and experiences as individuals, their relationships and interactions with others in their communities, and access to services. Individual written and verbal consent at the start of the interview process was used for adolescents aged 18 and 19. Younger adolescents, aged 15–17, assented and was supplemented by signed parental/guardian consent and verbal approval given during parent/guardian sessions. To maintain confidentiality, we attribute quotations with pseudonyms and some of the details of those whose cases are included in this article have been changed.

### Data collection

From 2015, the first author volunteered part-time at the teen club, and from November 2018 to June 2019, collected data over 8 months. A closed-ended survey was conducted with 406 ALHIV using the CYRM-28 survey to gather socio-demographic details and information on their access and utilisation of health and social services. Twenty-six in-depth interviews (IDIs) were then conducted, spread out across the months of fieldwork. Five of the 26 IDIs were scheduled by phone call within the week, and the investigator invited the adolescents to suggest a place of their choice, with the time chosen to build rapport and maintain confidentiality. Sometimes we sat in or outside of an adolescent’s home, at the netball or soccer grounds at their school, or in my makeshift office that I had decorated to provide an accessible and comfortable space in which young people could relax and talk freely. The next two rounds of the interviews with 10 adolescents were held in my makeshift office, where we reflected on the narratives and responses from the IDIs, while focusing on their own experiences at the teen club and with other service providers.

We accessed the participants’ clinic files and health passports (personal health cards) to capture their history of illness, including infections co-occurrent with HIV. In presenting each case study, we include viral load results and examples of illnesses as indications of the severity of HIV-related issues, adherence to medical treatment, service-seeking histories and provision of health care and support. Finally, 20 of the 26 adolescents participated in an ecomapping exercise, drawing their families and the resources they had used in the past year. Using a pen or pencil and paper to graphically represent people or services in their lives, such as friends, school relationships, relatives, doctors, neighbours and church members, these data helped us to triangulate our findings on service usage and relational supports, and strengthened our findings on how structural factors (gender, social and health services) affected young people’s narratives. To maintain consistency and trustworthiness, all interviews were conducted by the same person (first author), were digitally recorded, and lasted 45–60 min. Bottled water, soft drinks and buns were provided during breaks in interviews. Study participants were reimbursed transport costs at the end of each interview.

### Data analysis

Data analysis was phased. In the first phase, we summarised ALHIV socio-demographics, quantity of service use and clinical outcomes using descriptive statistics. Table 2 summarises their socio-demographic data and backgrounds. The discursive analysis of the drawings (ecomaps) with participants identified key experiences and themes [45, 46]. These ecomaps and themes were linked to data from IDIs to strengthen each case [47]. Framework analysis was used to make sense of the IDIs, notes from the adolescents’ clinic files, health passports, and field notes. All data were typed into Word and imported into NVIVO 11 to allow us to code all paragraphs and categories that showed multiple vulnerabilities and services.

Data were indexed along in three tiers—individual, relational and contextual—to highlight the complexity of young peoples’ lives. We noted all details related to lifetime or life history and teen-club clinic experiences, including knowledge of HIV status, disclosure processes, taking ART, friendships, family, love and sexual relationships, pregnancy, service usage, STI treatment processes, education services, labour ward experiences, and if relevant, interactions with correctional services. Charting allowed for themes within and between cases to be summarised. See Table 1 presents the main themes that emerged from the data according to tier. In this article, we have selected the life experiences of four participants to represent the main findings.

**Table 1:** Main themes for ALHIV life experiences

Individual	Relational	Contextual factors
Age	Parental/guardian interactions	Institutional support
HIV status	Death of single or both parents	Educational services
Gender	Fear of disclosure	HIV clinical and psychosocial services
School status	Lack of affective relationships	Clinic teen club
Premarital status	Peer pressure	Spiritual and herbal support
Strategic silence	Strategic silence	Safe spaces where adolescents learn

## Results

### Case studies

The four young people were aged 15––19, two young men who we call Timveni and Widze, and two young women, Chiletso and Chifuno. The experiences of Timveni and Widze indicate the influence of ideas of masculinity on service usage and relational experiences. Chiletso and Chifuno were both out of school, and their accounts emphasise their need to be accepted and how this influences their reproductive lives. Table 2 summarises their backgrounds.

**Table 2:** ALHIV case study participants

Pseudonym	Age	Gender	Level of education	Present occupation	Background notes
Timveni	17	Male	College	Student	Both parents passed away; alcohol misuse; multiple sexual partners; mouth ulcers; STIs
Chiletso	18	Female	Primary school	Small-scale business	Both parents passed away; ran away from home; pregnant at 14; living alone with a four-year-old child; transactional sex; STIs
Chifuno	19	Female	Primary school	Primary school drop out	Out of school; learning difficulties; unprotected sex; non-disclosure to partner; living at home with a single parent.
Widze	15	Male	Primary school	Student	Questioned by police twice; juvenile detention services twice; poor adherence to ART; tuberculosis

These four young people illustrate and explore the totality and complexity of adolescents struggles in different spaces. These four ALHIV all attended at least some teen-club clinic meetings, utilised alternative health facility services, and reported that they were doing well [36, 48]. But their divergent experiences heightened the complexity of information that emerged from the reviewed clinical files, health passports, and ecomap drawings of ALHIV, the latter identifying various medical and social resources. The in-depth interviews relayed the complexity of their medical histories, friendships, and relationships, and factors that resulted in social inclusion or exclusion. Their experiences provide the basis for our comprehensive analytical conclusion [30, 42].

#### Timveni

Timveni, or Timve as he preferred to be called, lives in what people locally refer to as a ‘ghetto’ (slum) in Blantyre, the commercial city in Malawi. We used to meet in the ART clinic corridors and exchange pleasantries, and sometimes he would walk me to my office, 2 km away. When completing our initial survey, Timve had requested psychological support, and I was mindful of his circumstances. We first met for an interview at his school. The second time we met at his home, and the third time at my office. As we began to talk for longer periods, Timve spoke of the value he placed on having me listen to the ‘pain’ in his life:

Each house I have lived in has different morals and beliefs, and it becomes difficult to change my behaviour every time I change houses. I have changed households more than four times. Sometimes I am forced to do work for other people. At the age of 8, I was sickly, my dad took my brother and me to a herbalist to get medicine to strengthen our immune system. Six months later, my health had deteriorated. A close friend of my dad drove my brother and me 400 km away from Blantyre to get tested and start antiretroviral treatment. In the same year, I lost my dad. My mom died a year later.

With both of his parents deceased, Timve’s brother is the only close family member that he has. He remains deeply unhappy about his mother’s untimely death. He remembers her telling him that life was not easy, especially when you take ARVs because it’s a lifelong task. He reported that he sometimes feels angry and depressed: “I need therapy. Sometimes I feel mentally insane and feel like nobody listens to me.” Timve has stayed at intervals with different aunts and uncles, but his relationship with them is difficult and has led to resentment. The aunt and uncle with whom he was living when we spoke provide him with food and shelter, but he believes they do not understand him and do not love him and he yearns for compassion and touch. Timve feels excluded from the interactions of others in the only home he has known for the past 5 years, especially after his aunt disclosed his status to neighbours. He also feels rejection by and alienated from others in the community.

Timve’s grief and loneliness have led to his use of alcohol and sex, and he yearns for romantic relationships. His sexual debut occurred when he was 11 years old. He had joined a gang in his neighbourhood who taught him how to pay for sex at guesthouses. He is embarrassed when he reflects on this in the interviews, and claims that he has no control over his sexual feelings. The girls replace his mother, who he misses deeply. When he is with a girl, he feels cared for and safe. “All I need is a hug. Some warmth… you know what I mean.” His girlfriend of the moment, of his own age, provides him with affection, and he has stopped having multiple relationships because the teen club discourages this.

Timve explains that he does not look for sexual intimacy, but that most of the girls he dates encourage him to sleep with them: he consummates the relationship as a sign of trust and love for the girl. For Timve as for other adolescents in this study, there was little transparency among young people about the nature of any romantic relationships, and poor communication about protection. Timve does not remember the number of times he has been involved in unprotected sex, and although he fears girls becoming pregnant, he uses condoms inconsistently. He has disclosed his HIV status to a few, but not all, girls, but he also emphasises that those to whom he has disclosed did not request that he use a condom. Timve gets condoms from the toilets or the reception area at the teen club, but he does not like these as they are thick and not perfumed. From the age of 15, Timve has had various part-time jobs, providing him with money to buy expensive condoms that are soft, perfumed, and relatively easy to use.

**Fig. 1:**
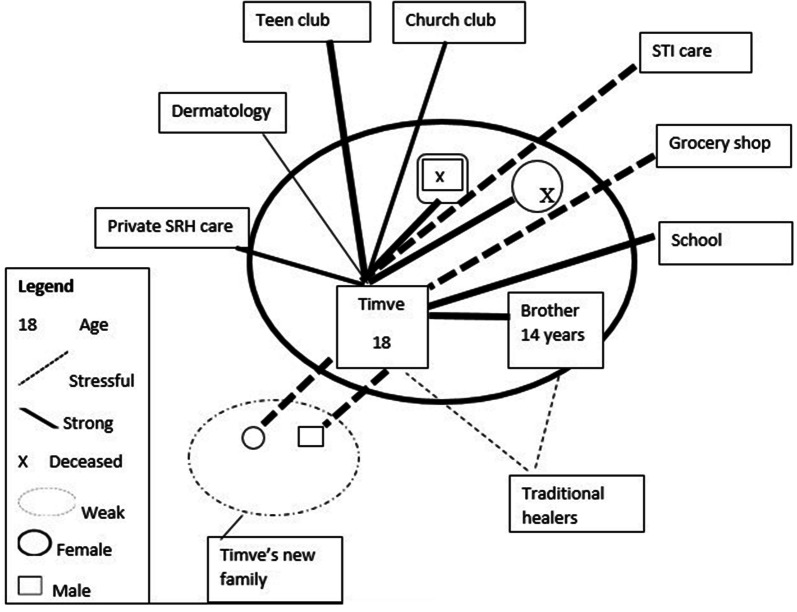
Timve’s ecomap. (Adapted from Hartman [78])

Timve attends the teen-club clinic to please his aunt. Even so, he has attended all scheduled teen club meetings for the past 2 years, and it is, he says, one of the few places where he feels alive; he laughs a lot. His health passport shows that his viral load was below the detection limit as determined in dry blood spot samples (commonly reported as ˂ 839 copies/ml by the local laboratory) in December 2018, suggesting good adherence to ART. According to contemporary WHO guidelines, a viral load below 1000 copies/ml was considered “virologically suppressed" [49]). He has had other health problems, however, including mouth ulcers and skin lesions. The sores in his mouth affect his smile since he fears being stigmatised, and he has visited Lepra (the hospital’s Dermatology Department), a 5-min walk from the teen-club, for treatment. He also attends a private clinic for SRH-related services when he gets STIs; he is embarrassed to talk about this. His ecomap (Fig. 1) depicts the relational and service support he has used in the past year.

Timve also spends time at church to please his aunt, and he refers to his endless search for connection and love at the teen-club at the church. He pays attention to his dress and personal presentation at church and teen meetings to demonstrate respect for the family and membership. He also seems confident; he argues that he has the potential to become someone in his life. People surrounding him acknowledge that he is intelligent. He is studying for a bachelor’s degree in mathematics and he is optimistic about this. He promises to work hard in his life, at school and in part-time jobs, so his brother (for whom he feels responsible) can move with him into a rented house.

#### Chiletso

We always met at Chiletso’s home, which was a bedsitter in a high-density area of Blantyre. Our meetings took place on Sunday afternoons after church. Chiletso is devout; she maintains her church calendar with weekly prayer meetings, including those that she hosts intermittently at her home. She believes in God as all-powerful, and that God has made provisions for her in the form of food, shelter, and protection in her solitary life. We shared roasted maize and sweet potato for tea whenever we met. Chiletso is grateful because the teen-club clinic provides space for her to talk about her life and needs. The club offers counselling sessions and an opportunity to have social relationships with others. Chiletso and her grandmother were referred to the club in 2010, when Chiletso was 8 years old and had been discharged from the central hospital. Chiletso was born with HIV “like mother-to-child transmission,” and she gets her medication from the teen club. She is happy, despite complaining that she is poor and blaming herself for dropping out of school; she refused to tell me her last grade. She dreams of becoming self-reliant. Her dreams for a better future led her to start a cross-border business, bulk buying juices from Zambia to sell in Malawi to meet her everyday needs.

**Fig. 2:**
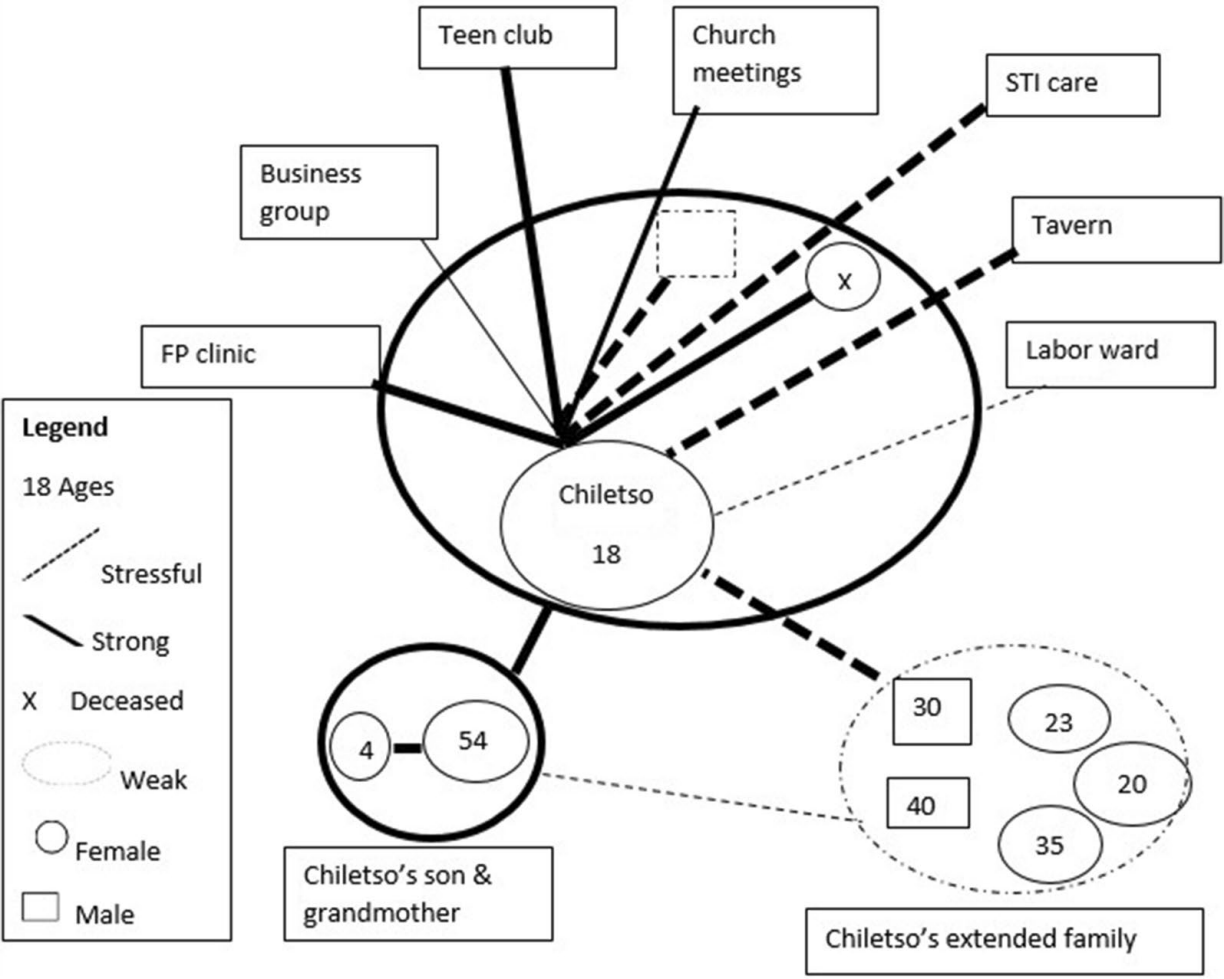
Chiletso’s ecomap. December 2018 (Adapted from Hartman [78])

Chiletso’s mother had died, although sometimes she claims that she is a “double orphan” since she does not know her father. She has strong family support. She loves her grandmother, aunts and uncles, and is very proud that they help her when she is in need. However, she prefers to live alone and has done so since she ran away from home at 16, because her grandmother kept telling her to get help from her dead mother at the graveyard. Tears swell up in Chiletso’s eyes when she talks about her mother. She wishes she were alive. Chiletso attends counselling with club mentors as part of the therapeutic sessions organised by teen club facilitators (Fig. 2).

It took four meetings with Chiletso before she was willing to discuss the main reason she had run away from home. That Sunday afternoon in February 2019, she began to discuss how she became pregnant. She emphasised that she did not have a choice; she argued that no, 14-year-old chooses to become a mother. She said she did not know much about contraception, but claimed that she became pregnant so that she could get help. She did not consider abortion because she regarded her pregnancy as fate. Her mother had not aborted her. She kept the pregnancy secret until she was in labour. Chiletso reminded me that one of the reasons she frequents the teen club is to find space to talk freely and get easy access to “this,” pointing to where Norplant had been inserted by one of the nurses at the teen-club. I acknowledged the advantages of the ‘bundled’ services or a ‘one-stop-shop’ at the teen club that gave young people access to both ART care and contraception.

Chiletso did not breastfeed her son because she had sores everywhere. Her medical records show that she had reacted badly to ART medication and that had caused the skin reaction. While her skin reaction was disfiguring, she insists that she will always adhere to ART medication because now it helps her to keep her skin beautiful. Her viral load continues to be below the detection limit in dry blood spot samples. Her grandmother, who lives in a village, came to Blantyre and took away her child. Chiletso maintains she would not have survived this long if she had been left alone to take care of her son. Extended family members buffer the impact of adolescents’ pregnancies.

Chiletso becomes sad when she talks about becoming what she calls a ‘child prostitute’ to survive and pay her rent. Sometimes she fails to make the US $10 to pay rent for a month, and she is evicted by her landlord. She tries to stay with friends when she has no cash but some, at least, are reluctant to accommodate her because they are afraid she might infect them; the stigma and discrimination associated with her HIV status is the main reason she lives alone and strives to do well. She hopes to get a partner and start a family soon, but she will wait patiently to marry someone who will fully accept her. She is willing to delay disclosing her status until she is sure she can trust her partner.

#### Chifuno

Chifuno likes to call herself my sister. Whenever we met at my make-shift office, she remarked that we looked alike. Chifuno maintains that she excelled at school, but after being in and out of the hospital a couple of times, her performance weakened. She began repeating classes and experiencing learning difficulties. She is frustrated because most friends with whom she was in primary school are now in high school; she has now repeated grade 5 three times. Even worse, some teachers have suggested that she is too old to be in primary school, and this annoys her because opportunistic infections due to HIV have delayed her progress. She decided to drop out of school as she prepared to start grade six. She mentions that she does not like reading anymore and she is functionally illiterate in both English and Chichewa. She wants to start a business, either opening a salon or making and selling duvets with the help of her mother, a single parent. However, 2 years after leaving school she is currently at home and is not working.

Chifuno found out that she was HIV positive two years before the interview. From 2015 to May 2017, she had poor viral load results (74,000 copies/ml), frequent periods of hospitalisation, and ART failure. The ART clinic switched her medication and her viral load is now suppressed below the detection limit in dry blood spot samples.

I thought going to the hospital when one is ill, is normal. However, I was surprised by how often I had to be taken to the hospital and the fact that I was the only child in the house who was frequently taken to the hospital. By the time I was able to read and see all those posters at the hospital, I realised I was HIV positive.

Chifuno’s grandmother continually monitors and counsels her about her health. She also periodically attends the teen club, although she is now 18 and feels she is too old to do so; the teen-club clinic is open to young people who have disclosed their HIV status and are aged 8–24. Chifuno cannot relate to younger members. Separate group meetings are held depending on age and sub-group, defined by colour [grey, green, yellow, blue, orange], to which they belong. The older teens (20–24 years) meet once every quarter of a year, and Chifuno identifies with this older group.

Chifuno lives with her biological mother but she has not disclosed her status to her. She does not talk much with her mother, who she says is “difficult.” Her mother has been married twice. Chifuno has no contact with her father, and her family do not talk openly about him. Some say he is in South Africa; her aunt says he died. Secrecy and conflict affect disclosure in the family. However, Chifuno mentions that being in love “keeps her alive.” She spends most of the time with her older boyfriend at her grandmother’s house. Although they were not formally introduced, both families know about the relationship and have accepted it. Chifuno enjoys sexual intimacy; while they used contraception initially, now they have unprotected sex and she looks forward to now becoming pregnant. She is letting nature take its course in her life and love life.

ALHIV strive to manage their lives through “strategic silence,” consciously and purposefully avoiding disclosure of their status to avoid the anxiety and emotional distress they experienced when first advised of their status. Chifuno has not disclosed her status to her boyfriend. She neither thinks about infecting her partner or being re-infected, nor does she think about STIs. She says she is playing the role of a fiancée, and speaks of being “promised to marry,” the local idiom adolescents use to refer to a serious relationship. Hence, she defers to her boyfriend to make all decisions. He is very argumentative, and as a result, she does not ask him to use protection as she fears he might get offended or accuse her of infidelity. She has tried to encourage him to go for HIV testing services, without seeming pushy, as she does not want to raise suspicions about her own status. She explains that she is waiting patiently for him to suggest that they visit the health centre and get tested and counselled together. Good and accessible health services are close to her home; but to avoid exposure and maintain her privacy, she goes to the teen-club clinic get her medication.

#### Widze

I met Widze, a 15-year-old, in the adolescents’ “back to care” office. The back-to-care team is a mentoring team that follows up adolescents who miss ART or teen club sessions; one of the team had contacted Widze because he had defaulted from care. His clinical records also showed that he had had tuberculosis in 2018. I gave him an invitation letter and an assent form to give to his guardian, and asked him if we could meet the following week at my office. Widze is the last born in a family of four. He refuses to talk about his parents, who died 6 years ago. Widze believes that his brothers and sister do not love him, and they do not want him to live with them. He feels like a ‘nobody’ in their home. Widze blames his brothers and sister for not disclosing his HIV status to him; they had never explained why he had to take medication every day for life. However, he acknowledges that his older brother introduced him to the teen club; he had attended it once.

Widze’s brother, who is his guardian, reported him to the police twice, and Widze has been questioned and retained by juvenile correction services twice. The first time the police detained him was because he beat up the man who was undertaking domestic work for his brother. He argued that he was not in the wrong because the man had disrespected him. He refused to talk about why he was sent to juvenile services the second time. He is angry and hurt that his brother reported him to the police and that, as a result, he had to spend time in the correctional service without his medication. This was evidence that his family wished him to end his life prematurely.

Widze has few friends and he says little about them; he describes himself as a loner. Yet despite this, his unhappiness at home, and his brushes with the law, he is a confident young man. He loves school, is hardworking, and speaks eloquently. He prefers to spend time with his teachers, who he believes wants the best for him. He attends special part-time classes without charge and receives special attention, books and support because the teachers know he is an orphan who needs extra support. When he disappears from school and presents to juvenile services, he mentions that he wishes he could go back and stay at the day school and not in his brother’s home because his teachers do not question his absenteeism:

Sometimes, I am away for two weeks. My teacher will not ask me too many questions. He encourages me not to miss school and helps me to catch up during part-time lessons. I do not pay for those lessons. That teacher cares for me.

Widze was reported as defaulting by the teen club tracing team, and was followed up. His viral load results were initially poor above 1000 copies/ml, in January 2019. The day he came to the back-to-care room, he explained his absence as due to his busy schedule at school and that he lacked the time to attend ART clinic. He promised to become a regular attendee at the teen club, but I have yet to meet him in the corridors of the teen club and we never had the chance to talk again. His medical records show that he finished his TB dosages, and the most recent viral load results were suppressed below the detection limit in dry blood spot samples by June 2019.

## Discussion

HIV acts as an entry point to services and improved knowledge on adherence, treatment-seeking, and relational support for adolescents and young adults during various life transitions. The four case studies presented above illustrate the complexity of the social lives and health needs of ALHIV in their homes, at school, within the community and at the ART and teen-club clinic settings. The main themes of their stories reflected interaction within these four ecologies and showed conflicts, affective peer and mentor support, individual risks, the breakdown of affective relationships, institutional support, and adolescents striving to do well despite their adversity.

Widze, Chifuno, Timve and Chiletso all have complex needs. However, partial attendance at the teen club supported their adherence to ART medication resulting in undetectable viral loads. While an undetectable viral load does not mean that a person’s HIV is cured, it does offer tremendous promise for their overall health, wellbeing and reduced risk of viral transmission to others. Findings in our study establish a counter-narrative for ALHIV of positive decision-making and meaning-making, including where and how they seek help as they strive for wellbeing. It is imperative to look at the context and culture-specific aspects of ALHIV ‘doing well’ by negotiating intimacy, peer and institutional support, love, safety and connectedness [10, 24, 28, 50].

Individual factors such as age, gender, level of schooling and pre-marital relationships have been thoroughly discussed in the literature as key determinants that affect adolescents’ health and social outcomes [18, 23, 34, 51–53]. While social determinants may reify the familiar dominant narrative of the risk discourse, ALHIV having increased mental health problems and lag in adherence, contraception use, disclosure, and viral load [10, 25, 28, 54–59]. In this article, the adolescents had been involved in sexual relationships at a young age but strive to protect themselves. An increasing number of young men are involved in casual sex with older women and girls within their age groups in Blantyre. In adolescent health, both girls’ and boys’ perspectives matter.

In our study, adolescent defaulters who had a poor viral load and were not attending the ART clinic were followed up and brought back to care. Providing one-to-one sessions with Widze offered him space within the adolescent back-to-care room to discuss his life experiences with his mentors. Widze was able to talk about the challenges he was facing at home, which affected his attendance at the ART and teen-club clinic. The presence of trusted back-to-care tracers, coupled with a conducive environment, encouraged him to openly express his feelings. The youth wellbeing policy review for Malawi mentions that support from friends and family encourages adolescents’ participation and empowerment [18, 25, 26]. Van Breda and Theron [36] also note that affective support from various groups of people a ‘supportive social ecology’ is key to successful social and health outcomes. Hence the relational factors in the home and at institutions are crucial for ALHIV’s social and health-related outcomes.

However, we found mixed responses from participants about the role of friends and family to encourage wellbeing and handle health-related shocks, in particular, disclosure and discrimination. For instance, Timveni and Chiletso experienced difficulties interacting with their uncles and aunts because their family members had told others of their HIV status. Denial, secrecy, child–parent conflicts, stigma and discrimination all affected young people’s understanding of living with HIV [58, 60, 61]. Through the two ecomaps and case study narratives, we learn that extended family members and friends could be father/mother figureheads and best friends to adolescents, but they were not entirely trusted.

ALHIV feared that because their adoptive guardians/caregivers were not responsible for their infection, they felt no constraint in telling others of their [the adolescents’] HIV status. Mandalazi and colleagues, who studied adolescents who were perinatally infected in Lilongwe, also found that adoptive guardians felt relatively free to disclose the adolescent’s status, because they were not responsible for or answerable as the source of an adolescent’s HIV infection [58]. This contrasts with van Breda and colleagues, who have argued that among a general population of adolescents, the most prominent supportive relationships were friends, parents and caregivers, and teachers [36]. Betancourt and colleagues, in a review of mental health, resilience and HIV, stated that disclosure and openness were related to resilience and self-efficacy, while silence, secrecy, and stigma contributed to feelings of self-hate, anxiety, hopelessness, and confusion among HIV/AIDS-affected children [6]. However, ALHIV in our study did not feel well supported, and hence they were reluctant to disclose their status. They resorted to ‘strategic silence’ as opposed to disclosure as an opportunity cost for personal and family wellbeing [9] and to support their resilience.

Religion played an influential role for two people presented above. Timveni went to church as a means of maintaining connectedness with family and church. Religion encouraged Chiletso to lead a stable life, knowing she was protected at all times. Ashaba and colleagues likewise found that religion offered ALHIV hope for the future in rural Uganda [62]. Attending church or a mosque provides religious support that can strengthen individual and group networks [36, 63].

Despite health facilities and governments trying to ‘normalize’ HIV by the routinization of testing, diagnosis and treatment, through the ‘test and treat policy’ of patient-centred care, for ALHIV, the disease remains exceptional [64, 65]. Chiletso’s, Timve’s and Chifuno’s accounts illustrate SRH-related challenges including negative emotional outcomes such as regret and physical health outcomes—STIs and pregnancy [11, 66, 67]. We see the responsibility of institutions to meet adolescents’ needs. ALHIV become involved in casual sex to gain a sense of affection and belonging, and for some, as a means of survival. To avoid unwanted disclosure, they do not request or insist on protection during sex. This is in line with findings from a systematic review of being on ART in Africa, which indicated that competition for relationships, material and marital responsibilities lead adolescents to difficulties to access support [15]. There is need to balance adolescents’ sexual interests and the rules defining sexual relationships to enable a negotiation space that allows for choices (delayed sex, less unsafe sex, contraception use, sexual knowledge) and informed consent.

For Chiletso, having a child ‘normalizes’ sexual activities and pregnancy outcomes [26, 54, 68]. In addition, having a child in some African societies is seen as enhancing both male and female identities as fathers or mothers [26, 69]. However, Chiletso gave birth at a very young age. She now uses contraception and plans to do so until she considers that she is ready for another pregnancy. Unintended and/or early pregnancy reinforces the need to continue sexual education and learning among ALHIV. The teen-club model as a ‘one-stop shop’ tries to cater to medical, social, relational, SRH and psychosocial support [25, 54, 70, 71]. This contrasts with most studies that view provider-adolescent relationships as hindering communication about sensitive issues (mental issues, sex, pregnancy, STIs), leading to poor health outcomes [26, 70]. Reflecting on how ALHIV talk and experience sex suggests changes in the experience of sex among young people, and both young men and women openly talked about love, intimacy, safer sex, contraception and marriage in the teen club in Blantyre.

Chiletso and Timve refer to their need for a psychologist to listen to their pain and help them to deal with rejection. Widze sought TB care and adhered to treatment until he was cured. Chifuno intermittently attends the teen club despite her concern that she might be too old to do so. The teen club provides a space for adolescents to walk in at any time to access counselling, and empowers them to let their needs to be known. The participatory approach that ALHIV take to maintain their health and wellbeing confirms that both young men and young women have risk profiles that need continuous support over time [71]. The success of the teen club shows the importance of a one-stop shop with the availability of mentors and health providers to monitor and protect young people. A key recommendations is to improve the integration of service provision and care of ALHIV [64, 72, 73]. As Grimrud and colleagues quote Cynthia Silvia Parker, Interaction Institute for Social Change, “(i)t is not about everybody getting the same thing. It is about everybody getting what they need in order to improve the quality of their[adolescent] situation” [72].

Despite the lack of coordinated care, the four adolescents discussed in this article strived to do well despite adversity. A characteristic feature of all four is that they recognised their challenges and were motivated to do better. Both young men aspired to improve their socioeconomic status through education: Widze continued to go to school despite being in and out of the juvenile centres; Timve worked hard to get a degree. Both young women continued to dream of becoming businesswomen despite their learning difficulties. They also both sought knowledge on contraception. Chifuno cherishes her love relations and considers using services closer to her home to avoid transport costs. Liborio and Ungar [74] argue that involving adolescents in work can be burdensome, but it also encourages a sense of hope, agency and respect from others for contributing to their wellbeing. The four adolescents discussed above continue to reflect on their lives and hardships and reframe positive goals related to their education, sexual and reproductive health, maturation and entrepreneurship to enhance their lives [75, 76].

## Limitations

The study was conducted with ALHIV attending an ART and teen-club clinics in urban Blantyre. The findings may be unique to the four ALHIV and cannot be generalised, although the four were selected because they illustrated themes identified by all study participants. By including two young men and two young women, we hoped to reduce gender bias and provide in-depth insights on complex needs for both genders. Despite the possibility of interviewer bias, the first author had volunteered in the teen club since 2015, and this may have encouraged the ALHIV to give ‘honest’ responses and talk freely with her.

## Conclusion and recommendations

ALHIV are a diverse group with needs in multiple areas. In this article we illustrate the value of attending to their diverse narratives as they strive for a better life, sense of autonomy, aspirations and improving health seeking behaviours and health outcomes. Adolescents, we note, act strategically. In order not to undermine their resilience and maintain the support system around them, they opt for silence concerning their HIV status.

The complex needs for young people can affect their service usage but can also motivate them to do well despite adversities [31]. While in Malawi, adolescent care at a country level is fragmented due to service specialisation, the teen-club clinic (as a one-stop shop) is a key resource that ALHIV recognise as providing them with space to learn and enrich their lives. The support from the teen-club clinic is not only clinical and motivational, encouraging adherence to ART and helping to suppress ALHIV’s viral load; health workers and mentors also address young people’s evolving needs and decision/meaning-making processes. Coordinated and integrated services prove to be valuable to meet the complex needs of ALHIV over time.

Social and relational resources are not just found; they are in part created by adolescents themselves. Ecomaps and case study narratives revealed that adoptive parents or guardians and friends were not fully part of a ‘social-ecological support’ network. The maps illustrated the role of the family structure in HIV care and support [58, 60], but how ALHIV interact, relate and discuss their health among themselves and with others is nuanced and strategic. Future studies among ALHIV should explore strategic silence and secrecy within relational, community and institutional supports, using longitudinal studies to understand how adolescents build and sustain relationships in various sociocultural contexts [55, 58, 77]. This approach is key to creating wellbeing or resilience frameworks to support ALHIV across sub-Saharan Africa.

## Data Availability

Data from this study will be made available upon request from Blessings Kaunda-Khangamwa at b.n.kaunda@gmail.com. If one needs to use the data, they will need to seek approval from the Lighthouse Trust and their review boards.
